# Enrichment of fetal and maternal long cell‐free DNA fragments from maternal plasma following DNA repair

**DOI:** 10.1002/pd.5406

**Published:** 2019-01-10

**Authors:** Joaquim S.L. Vong, Peiyong Jiang, Suk‐Hang Cheng, Wing‐Shan Lee, Jason C.H. Tsang, Tak‐Yeung Leung, K.C. Allen Chan, Rossa W.K. Chiu, Y.M. Dennis Lo

**Affiliations:** ^1^ Centre for Research Into Circulating Fetal Nucleic Acids, Li Ka Shing Institute of Health Sciences The Chinese University of Hong Kong Shatin, New Territories Hong Kong SAR China; ^2^ Department of Chemical Pathology The Chinese University of Hong Kong Shatin, New Territories Hong Kong SAR China; ^3^ Department of Obstetrics and Gynaecology The Chinese University of Hong Kong Shatin, New Territories Hong Kong SAR China

## Abstract

**Objective:**

Cell‐free DNA (cfDNA) fragments in maternal plasma contain DNA damage and may negatively impact the sensitivity of noninvasive prenatal testing (NIPT). However, some of these DNA damages are potentially reparable. We aimed to recover these damaged cfDNA molecules using PreCR DNA repair mix.

**Methods:**

cfDNA was extracted from 20 maternal plasma samples and was repaired and sequenced by the Illumina platform. Size profiles and fetal DNA fraction changes of repaired samples were characterized. Targeted sequencing of chromosome Y sequences was used to enrich fetal cfDNA molecules following repair. Single‐molecule real‐time (SMRT) sequencing platform was employed to characterize long (>250 bp) cfDNA molecules. NIPT of five trisomy 21 samples was performed.

**Results:**

Size profiles of repaired libraries were altered, with significantly increased long (>250 bp) cfDNA molecules. Single nucleotide polymorphism (SNP)‐based analyses showed that both fetal‐ and maternal‐derived cfDNA molecules were enriched by the repair. Fetal DNA fractions in maternal plasma showed a small but consistent (4.8%) increase, which were contributed by a higher increment of long fetal cfDNA molecules. *z*‐score values were improved in NIPT of all trisomy 21 samples.

**Conclusion:**

Plasma DNA repair recovers and enriches long cfDNA molecules of both fetal and maternal origins in maternal plasma.

## Abstract 2

What is already known about this topic?

- Most of the cell‐free DNA (cfDNA) fragments in maternal plasma have sizes less than 200 bp, with fetal molecules being shorter than maternal ones.
- DNA damages exist in cfDNA, particularly single‐strand nicks.
- Occasional no call for noninvasive prenatal testing (NIPT) can be caused by insufficient fetal DNA fraction.

What does this study add?

- Repair of cfDNA by PreCR repair mix can recover a subset of long (>250 bp) cfDNA molecules.
- Both fetal and maternal long cfDNA are enriched by PreCR repair treatment.
- Mild but consistent increments in fetal DNA fractions after PreCR repair, which are contributed by higher enrichment of long fetal cfDNA molecules.
- PreCR repair treatment improves NIPT of trisomy 21 by elevating *z* scores resulting in better discrimination of aneuploid from euploid samples.

## 1 INTRODUCTION

The detection of circulating cell‐free fetal DNA in maternal plasma has been increasingly adopted in noninvasive prenatal testing^1^ (NIPT) since its discovery in 1997. Cell‐free DNA (cfDNA) molecules of different origins have characteristic size distributions, with the most abundant 166‐bp peak for maternal cfDNA molecules and a relatively prominent 143‐bp peak for the fetal cfDNA molecules.^2^ Size differences between fetal and maternal cfDNA have been used for the development of size‐based NIPT.^2^, ^3^, ^4^ Fetal DNA fraction is a major contributor for successful NIPT. Estimation of the fetal DNA fraction has been shown to be achievable, even with shallow sequencing depths.^5^ Various approaches of fetal DNA fraction estimation have been developed^2^, ^4^, ^5^, ^6^, ^7^, ^8^, ^9^; some are male fetus specific,^4^, ^6^, ^7^, ^10^ while others are independent of fetal sex.^2^, ^5^, ^8^, ^9^ Despite the international adoption of NIPT, a number of issues need further refinements, including false‐negative and no‐call results.^11^, ^12^, ^13^, ^14^ Partly for these reasons, a number of approaches have been explored to enrich fetal DNA from maternal plasma.^3^, ^10^, ^15^, ^16^, ^17^, ^18^ However, some of these methods have their disadvantages, including the use of more complicated sample processing protocols, high unit cost, more labor intensive, and PCR bias.

DNA damages, for example, single‐strand nicks, exist and are present among cfDNA molecules.^19^, ^20^ Apoptosis, which is believed to be one of the major mechanisms of cfDNA production, involves DNA fragmentation during karyorrhexis, followed by the formation and production of apoptotic bodies.^21^, ^22^, ^23^ Commonly used sequencing library preparation methods employ the repair of DNA ends only, leading to the loss of damaged cfDNA fragments such as those with single‐strand nicks.^20^, ^24^, ^25^ Therefore, the detection of these damaged cfDNA molecules by the current paired‐end (PE) massively parallel sequencing (MPS) platforms is challenging.

To salvage the damaged DNA molecules, the use of PreCR repair mix, a commercial DNA repair kit, has been shown to be effective in recovering damaged DNA in forensics,^26^, ^27^ archeology,^28^ and molecular diagnostics.^29^ We hypothesized that the PreCR repair mix could also be used to repair cfDNA in maternal plasma. Repaired maternal plasma DNA with higher DNA integrity might enhance the success for downstream analysis. Due to the fact that fetal‐derived cfDNA molecules are shorter and more fragmented than the maternal ones,^2^, ^30^ we proposed that fetal cfDNA molecules possess more DNA damages. By repairing these damaged fetal cfDNA molecules, the fetal DNA fraction may be enriched.

In this study, we applied PreCR repair treatment on cfDNA from maternal plasma of first and third trimester pregnancies. We studied and compared the size profiles and fetal DNA fractions between repaired cfDNA samples and their sham controls. We also evaluated the impact of PreCR repair treatment on the performance of NIPT on samples collected from trisomy 21 pregnancies.

**What is already known about this topic?**

- Most of the cell‐free DNA (cfDNA) fragments in maternal plasma have sizes less than 200 bp, with fetal molecules being shorter than maternal ones.
- DNA damages exist in cfDNA, particularly single‐strand nicks.
- Occasional no call for noninvasive prenatal testing (NIPT) can be caused by insufficient fetal DNA fraction.

**What does this study add?**

- Repair of cfDNA by PreCR repair mix can recover a subset of long (>250 bp) cfDNA molecules.
- Both fetal and maternal long cfDNA are enriched by PreCR repair treatment.
- Mild but consistent increments in fetal DNA fractions after PreCR repair, which are contributed by higher enrichment of long fetal cfDNA molecules.
- PreCR repair treatment improves NIPT of trisomy 21 by elevating *z* scores resulting in better discrimination of aneuploid from euploid samples.

## 2 MATERIALS AND METHODS

### 2.1 Subjects, sample collection, and DNA extraction

This study was approved by the institutional research ethics committee. Pregnant women with singleton male fetuses were recruited from the Department of Obstetrics and Gynaecology, Prince of Wales Hospital, Hong Kong, with informed consent (Table S1). Plasma was isolated from 20 mL of EDTA‐anticoagulated maternal peripheral blood as previously described.^2^ For third trimester cases, 1 cm^3^ of placental tissue was dissected freshly after delivery from a region 2 cm deep and 5 cm away from the umbilical cord insertion. For first trimester cases, chorionic villus samples (CVS) were obtained. Plasma and buffy coat genomic DNA were extracted using the QIAamp DSP DNA Blood Mini Kit (Qiagen) and QIAamp DNA Blood Mini Kit (Qiagen), respectively, and quantified by Qubit 3.0 (Invitrogen). Placental DNA and chorionic villus DNA were extracted using the QIAamp DNA Mini Kit (Qiagen) according to manufacturer's protocols.

### 2.2 Plasma cfDNA repair and sequencing library preparation

Ten nanograms of extracted plasma DNA was subjected for DNA repair, using the PreCR repair mix (New England Biolabs). A sham control was done in parallel with the same amount of DNA input and reaction buffer only without the active enzymes. All DNA repair or sham reactions were done according to manufacturer's instructions. Sham‐ or repaired‐treated plasma DNA was purified by a MinElute Reaction Cleanup Kit (Qiagen) to remove residual enzymes and reagents, followed by PE sequencing library preparations. Double‐stranded DNA (dsDNA) libraries were generated as previously described.^31^ Libraries were quantified by Qubit dsDNA HS Assay Kit (Thermo Fisher Scientific) and real‐time quantitative PCR (KAPA Library Quantification Kit) on a LightCycler 96 System (Roche). The size profiles of the sequencing libraries were examined by Agilent 4200 TapeStation System with High Sensitivity D1000 ScreenTape (Agilent) to confirm successful library preparation before sequencing.

### 2.3 Targeted capture enrichment and MPS

Targeted capture of sequencing libraries was performed as described.^32^ The capture probes (Roche Nimblegen) predominantly targeted sequences on Chr6 and ChrY and were designed for a previous study on noninvasive prenatal assessment of congenital adrenal hyperplasia.^32^ Briefly, capture probes were designed to target a size of approximately 6‐Mb region, with each approximately 3 Mb flanking *CYP21A2* gene located on 6p21.3. For ChrY, a total size of approximately 1 Mb targeting ChrY unique regions was designed for detecting fetal signal in the maternal plasma. All repeat regions were excluded from the probe designing process. An average sequencing depth of 152 times per base (ranging from 82× to 210×) was obtained. Target capture enrichment efficiency was validated by quantitative real‐time PCR. All libraries were sequenced with a PE format of 75 bp × 2 on a NextSeq 500 System (Illumina). Adaptor sequences and low‐quality bases (ie, quality score less than five) were removed, and sequencing reads were aligned to the nonrepeat‐masked human reference genome (hg19) using the Short Oligonucleotide Alignment Program 2 (SOAP2).^33^ Up to two nucleotide mismatches but not indels were allowed for each member of the PE reads.

### 2.4 Single‐molecule real‐time sequencing

The pretarget captured sequencing libraries with Illumina format adapters were pooled and subjected to single‐molecule real‐time (SMRT) sequencing template construction using a SMRTbell Template Prep Kit 1.0—SPv3 (Pacific Biosciences). The posttarget captured Illumina sequencing libraries were similarly pooled and further subjected to SMRT sequencing library construction. The amplicon template preparation and sequencing protocol was used, with minor modifications: DNA was purified with 1.8× AMPure PB beads, and library size was estimated using a TapeStation instrument (Agilent). Sequencing primer annealing and polymerase binding conditions were calculated with the SMRT Link v5.1.0 software (Pacific Biosciences). Briefly, sequencing primer v3 was annealed to the sequencing template, and then polymerase was bound to templates using a Sequel Binding and Internal Control Kit 2.1 (Pacific Biosciences). Sequencing was performed on a Sequel SMRT Cell 1M v2. Sequencing movies were collected on the Sequel system for 10 hours with a Sequel Sequencing Kit 2.1 (Pacific Biosciences).

### 2.5 Microarray genotyping and single‐nucleotide polymorphism identification

Fetal and maternal genomic DNA samples were genotyped with the Infinium Omni2.5‐8 V1.3 Kit and the iScan System (Illumina). Single‐nucleotide polymorphisms (SNPs) were called by the Birdseed v2 algorithm with a confidence score cutoff^34^ of 0.15. The genotypes of the CVS and placentas were compared with those of the mothers to identify the fetal‐specific and maternal‐specific SNP alleles. An SNP was considered as fetal‐specific if it was homozygous in the mother and heterozygous in the fetus and the reverse for maternal‐specific SNPs. The fetal DNA fractions were deduced as described.^2^ Briefly, fetal DNA fraction (*F*) was deduced by the allelic ratio between a fetal specific SNP allele (*p*) and a common SNP allele (*q*) shared by the mother and the fetus using the following formula^5^:

$$F=\frac{2p}{p+q}\times 100\%.$$

In the size‐fractionated fetal fraction analysis, fetal DNA fragments were divided into 10‐bp bins as previously described,^31^ with modified size range of analysis. For detection of trisomy 21, overrepresentation of Chr21 of each sample was quantified by the *z* score using the following formula:

$$z\;\text{score}=\frac{\text{GRchr}21_{\text{test sample}}-\text{mean GRchr}21_{\text{euploid}}}{{\mathrm{SD}}_{\text{euploid}}},$$

where GRchr21 is the genomic representation (GR) of chromosome 21.

### 2.6 Statistical analysis

Student paired *t* tests and Wilcoxon signed rank test were performed using the International Business Machine (IBM) Statistical Package for Social Sciences (SPSS) Statistics (IBM). A *P* value of less than 0.05 was considered as statistically significant.

## 3 RESULTS

### 3.1 Size profile characteristics of sham‐ or PreCR‐repaired cfDNA from first and third trimester maternal plasma samples

**Figure 1:**
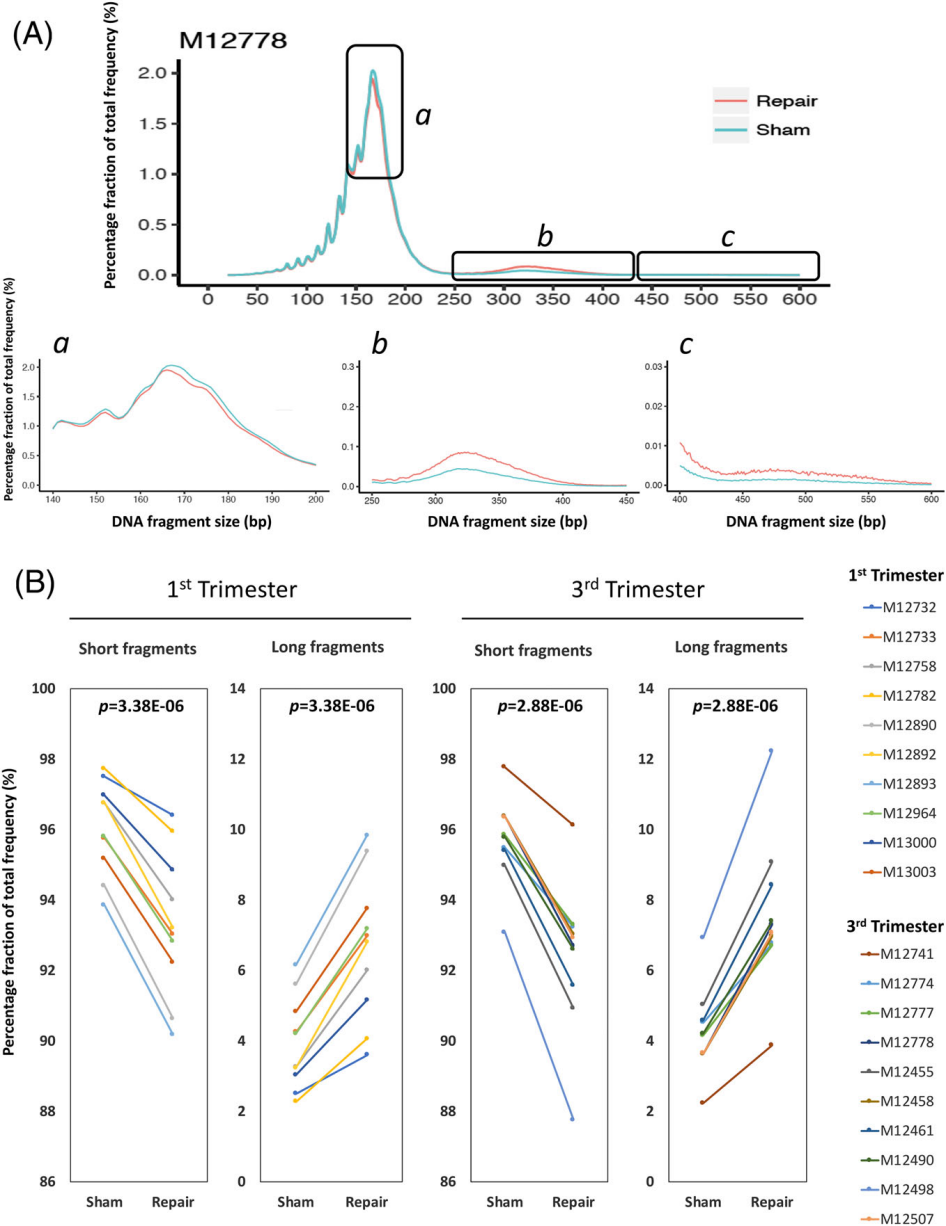
PreCR repair changes size profile of cell‐free DNA (cfDNA) in maternal plasma. A, Size profile comparison of sham‐repaired and PreCR‐repaired cfDNA from a representative third trimester maternal plasma (M12778). Lower panels (A‐C) represent the magnified size profiles of long cfDNA molecules (black boxes). B, Quantification of cfDNA molecules with different sizes in first and third trimester maternal plasma (10 samples each) after sham or PreCR repair treatment. There is a consistent decrease of short (0‐250 bp) and increase of long (251‐600 bp) cfDNA molecules after repair. Student paired *t* tests, first trimester's short and long fragments: both *P* = 3.38E‐06; third trimester's short and long fragments: both *P* = 2.88E‐06 [Colour figure can be viewed at wileyonlinelibrary.com]

**Figure 2:**
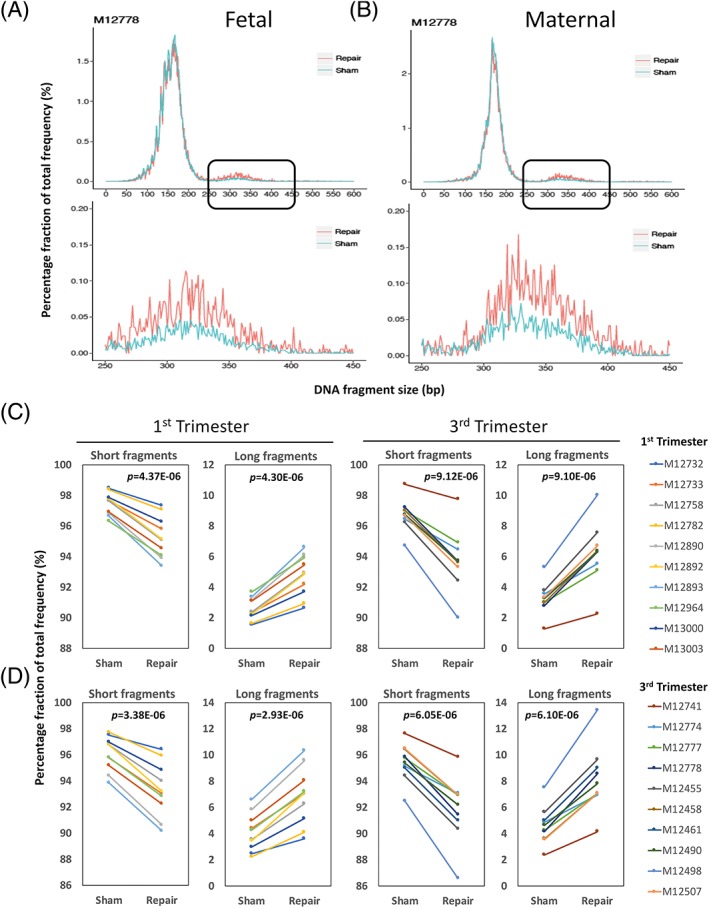
PreCR treatment repairs cell‐free DNA (cfDNA) molecules of both fetal and maternal origins. A,B, Averaged size profile comparison of sham‐ and PreCR‐repaired cfDNA from fetal (A) and maternal (B) cfDNA molecules. Lower panels represent the magnified size profiles of long cfDNA molecules (black box). C,D, Quantification of short (0‐250 bp) and long (251‐600 bp) cfDNA molecules in fetal (C) and maternal (D) cfDNA molecules from first and third trimester maternal plasma (10 samples each) after sham or PreCR repair treatment. Student paired *t* tests, first trimester long fragments: fetal (*P* = 4.30E‐06), maternal (*P* = 2.93E‐06); third trimester long fragments: fetal (*P* = 9.10E‐06), maternal (*P* = 6.10E‐06) [Colour figure can be viewed at wileyonlinelibrary.com]

We first studied if there was any size profile change of cfDNA in maternal plasma after sham or PreCR DNA repair treatment. Grossly, the size profiles between sham and repair groups did not show major differences within the range of 0 to 250 bp from sequencing. Both groups had a major peak size at around 166 bp and a series of peaks with 10‐bp periodicity from 70 to 150 bp (Figure 1A). However, we observed that there was an average of 3.2% decrease (95.9%‐92.9%) of short (0‐250 bp) cfDNA fragments and an average of 74.0% increase (4.1%‐7.1%) of long (251‐600 bp) cfDNA fragments in all samples after repair treatment, when compared with their sham counterparts (Figures 1A and S1). Statistical analyses showed that both changes were significant (both *P* = 1.85E‐11; Student paired *t* test). This trend was consistent when testing the first (2.9% decrease of short cfDNA fragments on average, 69.6% increase of long cfDNA fragments on average, both *P* = 3.38E‐06; Student paired *t* test) and third trimester samples (3.5% decrease of short cfDNA fragments on average, 78.1% increase of long cfDNA fragments on average, both *P* = 2.88E‐06; Student paired *t* test) (Figure 1B). We next asked if these size changes after repair treatment showed any relationship to the fetal or maternal origin of the cfDNA molecule. Using SNP‐based genotypes, we separated the cfDNA fragments of all subjects into their respective maternal and fetal origins. cfDNA fragments carrying fetal‐ or maternal‐specific SNP alleles were separated and considered as fetal‐ or maternal‐specific cfDNA molecules, respectively.^31^ We observed a similar trend in repaired cfDNA molecules from both fetal and maternal origins (Figures 2A,B and S2A). There was an average increase of 89.0% (2.9%‐5.5%) and 73.0% (4.3%‐7.5%) of long fetal and maternal cfDNA molecules in all samples after repair treatment, respectively (Figure 2C,D). Statistical analyses showed that both increments were significant (fetal: *P* = 2.80E‐10; maternal: *P* = 5.82E‐11; Student paired *t* test). When separated into first and third trimesters, the increase trends were similar. For first trimester samples, there was 85.5% increase of long fetal cfDNA fragments on average (*P* = 4.30E‐06; Student paired *t* test) and 68.3% increase of long maternal cfDNA fragments on average (*P* = 2.93E‐06; Student paired *t* test). For third trimester samples, there was 91.7% increase of long fetal cfDNA fragments on average (*P* = 9.10E‐06; Student paired *t* test) and 77.2% increase of long maternal cfDNA fragments on average (*P* = 6.10E‐06; Student paired *t* test) (Figures 2C,D and S2B). These results suggested that there was a relative higher enrichment of fetal long cfDNA molecules after repair, when compared with their maternal counterparts.

### 3.2 Fetal DNA fraction characteristics of sham‐ or PreCR‐repaired cfDNA libraries from first and third trimester maternal plasma samples

**Figure 3:**
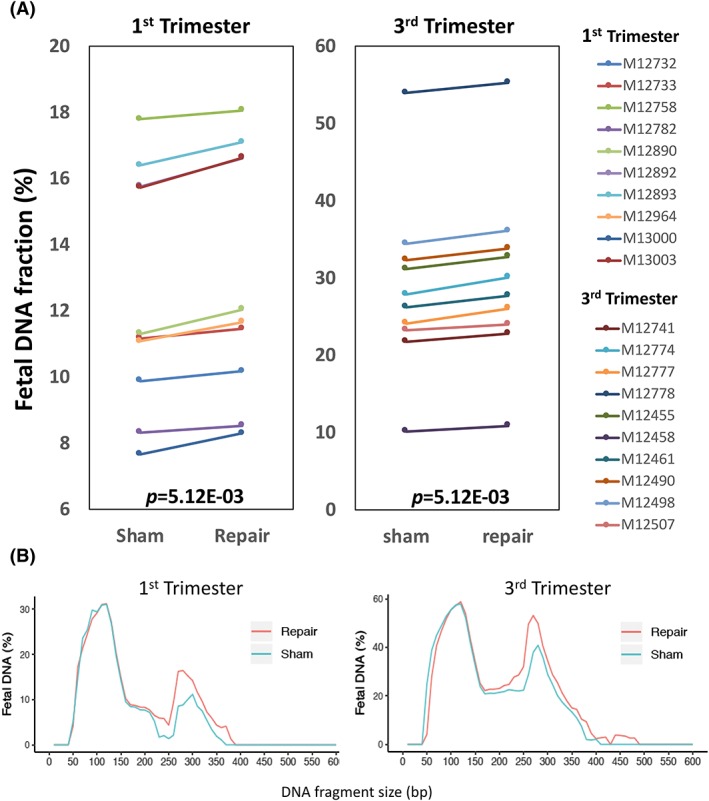
Fetal DNA fraction characterizations of PreCR‐repaired cfDNA. A, Fetal DNA fraction of sham‐ or PreCR‐repaired cfDNA from first and third trimester maternal plasma (10 samples each). Repair treatment gives a small but consistent fetal DNA fraction increase. Wilcoxon signed rank test, both *P* = 5.12E‐03. B, Size‐fractionated fetal DNA fractions of pooled first (left) and third (right) trimester maternal plasma cfDNA (10 samples each) after sham or PreCR repair treatment. There were mild increases in fetal DNA fractions among the long (approximately 300 bp) cfDNA molecules in the repaired groups [Colour figure can be viewed at wileyonlinelibrary.com]

As the fetal DNA fraction is an important parameter for successful NIPT, an increase in this metric might potentially benefit the sensitivity of NIPT, especially in cases with insufficient fetal contribution.^35^ Therefore, we characterized and compared the overall fetal DNA fractions of maternal plasma from first and third trimester samples after sham and PreCR repair treatments. We observed a consistent 4.4% increase of fetal DNA fraction (12.5%‐13.1%) for first trimester samples and 5% increase of fetal DNA fraction (28.5%‐30.0%) for third trimester samples after the PreCR repair treatment compared with their sham controls (Figure 3A). Statistical analysis showed that such increments were significant (both *P* = 5.12E‐03; Wilcoxon signed rank test). We speculated that the source of such mild fetal DNA fraction increment was contributed from the newly repaired fetal long cfDNA molecules. To prove this, we applied size‐fractionated fetal fraction analysis on both sham‐ and PreCR‐repaired subjects. Fetal DNA fragments were divided into 10‐bp bins, ranging from 0 to 600 bp. Fetal DNA fractions within each bin were then deduced separately.^31^ We observed that such fetal DNA fraction increment was mainly contributed by the increase of fetal cfDNA molecules with the size of around 300 bp (Figures 3B and S3). Taken together, these results suggested a higher enrichment of long fetal cfDNA molecules over their maternal counterparts in maternal plasma.

**Figure 4:**
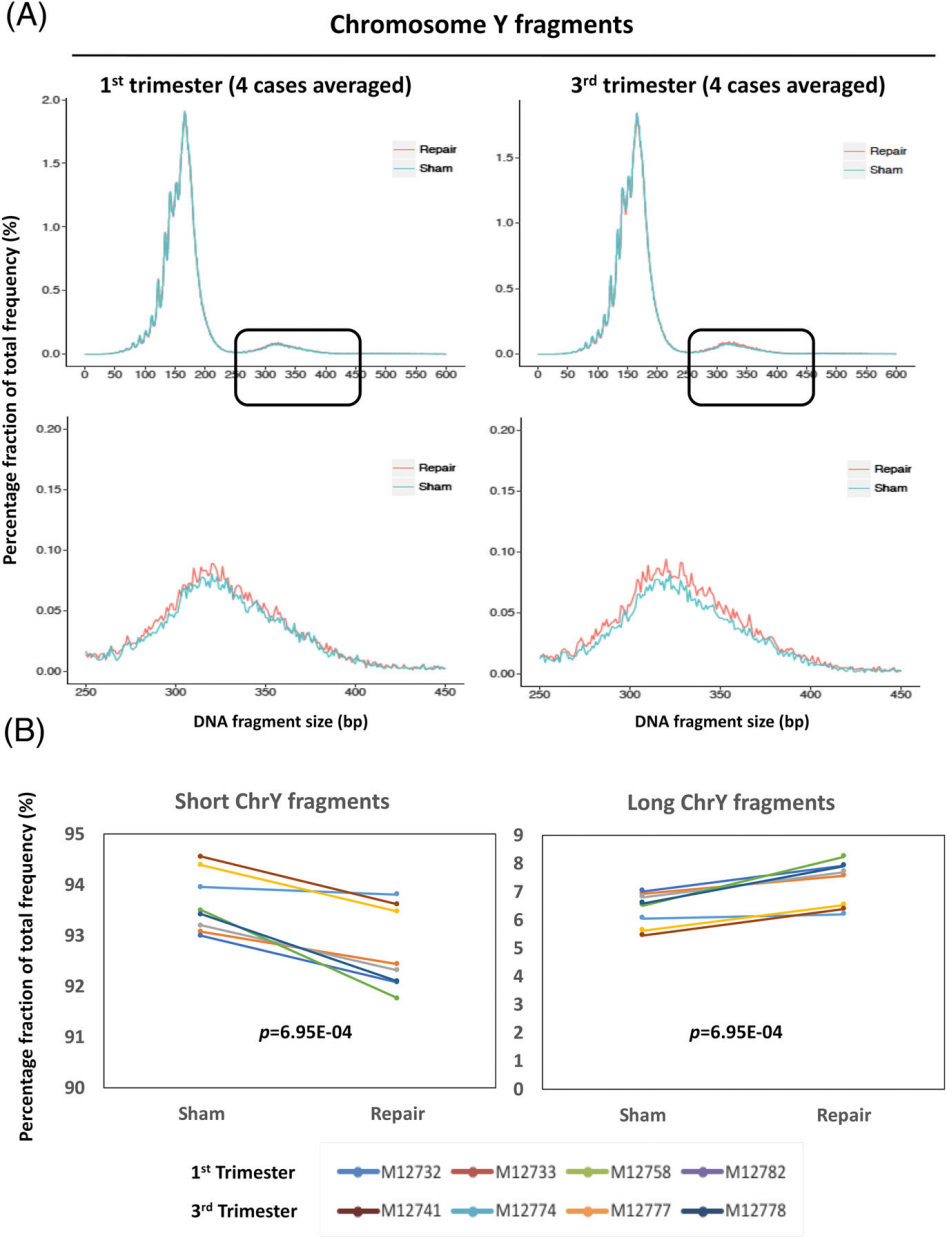
Increased sequencing depth of fetal cell‐free DNA (cfDNA) molecules by chromosome Y targeted capture. A, Averaged size profile comparison of sham‐ and PreCR‐repaired cfDNA molecules from chromosome Y in first (left) and third (right) trimester maternal plasma (four samples each). Lower panels represent the magnified size profiles of long cfDNA molecules (black box). B, Quantification of short (0‐250 bp) and long (251‐600 bp) cfDNA chromosome Y cfDNA molecules from first and third trimester maternal plasma (four samples each) after sham or PreCR repair treatment. Student paired *t* tests, *P* = 6.95E‐04 [Colour figure can be viewed at wileyonlinelibrary.com]

As fetal cfDNA molecules are the minority species in the maternal plasma, most of the data obtained from the total maternal plasma cfDNA sequencing are dominated by maternally derived cfDNA molecules. To obtain increased sequencing depth of fetal cfDNA molecules specifically, we used a target capture approach with probes hybridizing to sequences on Chr6 and ChrY.^32^ The capture probes on chr6 would target both the fetal and maternal cfDNA molecules while the probes on ChrY were designed to target only the fetal cfDNA molecules. From the combined Chr6‐ChrY target capture probes, we achieved an average sequencing depth of 152× per base for all sham‐ and PreCR‐repaired samples. The observation from the targeted sequencing results was concordant with the nontarget captured data, with a 13.6% enrichment (10.5%‐12.0%) of long cfDNA molecules from Chr6 and ChrY between 250 and 600 bp in size, after PreCR repair treatment (Figure S4). When counting only the captured fetal cfDNA molecules, we observed an average of 14.7% enrichment (6.4%‐7.3%) of long chromosome Y cfDNA molecules after PreCR repair treatment over their sham counterparts (Figure 4A,B). Statistical analyses showed that such increment was significant (*P* = 6.95E‐04; Student paired *t* test). Hence, we have demonstrated that PreCR repair treatment can recover a subset of long (>250 bp) cfDNA molecules in maternal plasma, both of fetal and maternal origins. The small but consistent increase of total fetal DNA fraction after PreCR repair was contributed by a higher enrichment of long fetal cfDNA molecules over their maternal counterparts.

### 3.3 Enrichment of cfDNA molecules longer than 250 bp from maternal plasma after PreCR repair treatment

**Figure 5:**
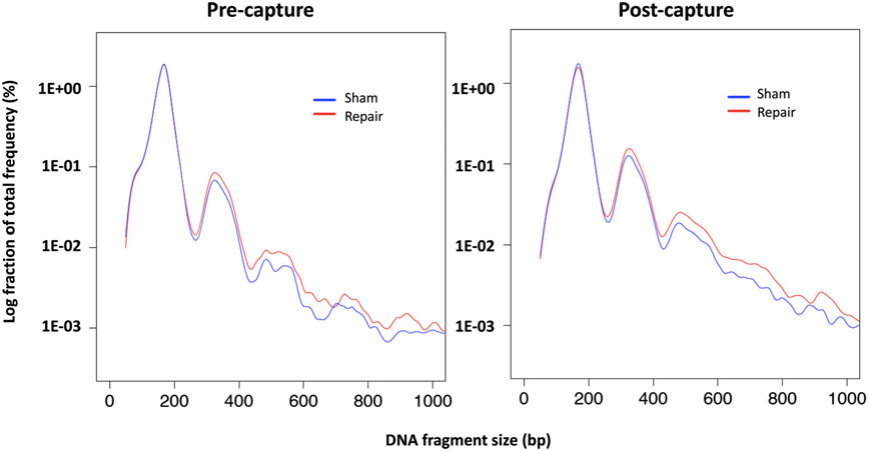
PreCR repair treatment enriches cfDNA molecules longer than 250 bp. Pooled samples of pretarget (left) and posttarget (right) captured libraries showed an enrichment of cfDNA molecules longer than 250 bp after PreCR repair treatment, with more prominent enrichment for molecules longer than 400 bp [Colour figure can be viewed at wileyonlinelibrary.com]

As the major size differences between sham and repair cfDNA resided on the cfDNA molecules longer than 250 bp, detection of such molecules by conventional next‐generation sequencing (NGS) platforms, such as the Illumina platform, might not be optimal due to their short read lengths. A third‐generation sequencing platform from Pacific Biosciences, which employed the SMRT technology, can achieve a sequencing read lengths exceeding 20 kb.^36^ We hypothesized that using such a technology, we might improve our ability to observe the cfDNA molecules longer than 250 bp in the maternal plasma. Due to the limitation of minimum cfDNA input, we pooled all Illumina‐formatted pretarget and posttarget captured libraries and resequenced them on SMRT sequencing platform rather than directly sequencing DNA template. We expected an enrichment of long cfDNA molecules after PreCR repair treatment. Consistent with our sequencing data from the Illumina platform, we observed a respective 31.3% and 28.9% enrichment of cfDNA molecules longer than 250 bp in pooled libraries with or without target capture after PreCR repair treatment (Figure 5). These enrichments were even more remarkable for cfDNA molecules longer than 600 bp (52.0% and 50.4% in pretarget and posttarget captured libraries, respectively) (Figure 5). These data not only further confirmed our previous observations of long cfDNA molecule enrichment after PreCR repair treatment in PE, shorted‐read sequencing platform but also shed light on a population of long cfDNA molecules that had not been analyzed by MPS in previous studies.

### 3.4 Improved NIPT performance of trisomy 21 after PreCR repair treatment

**Figure 6:**
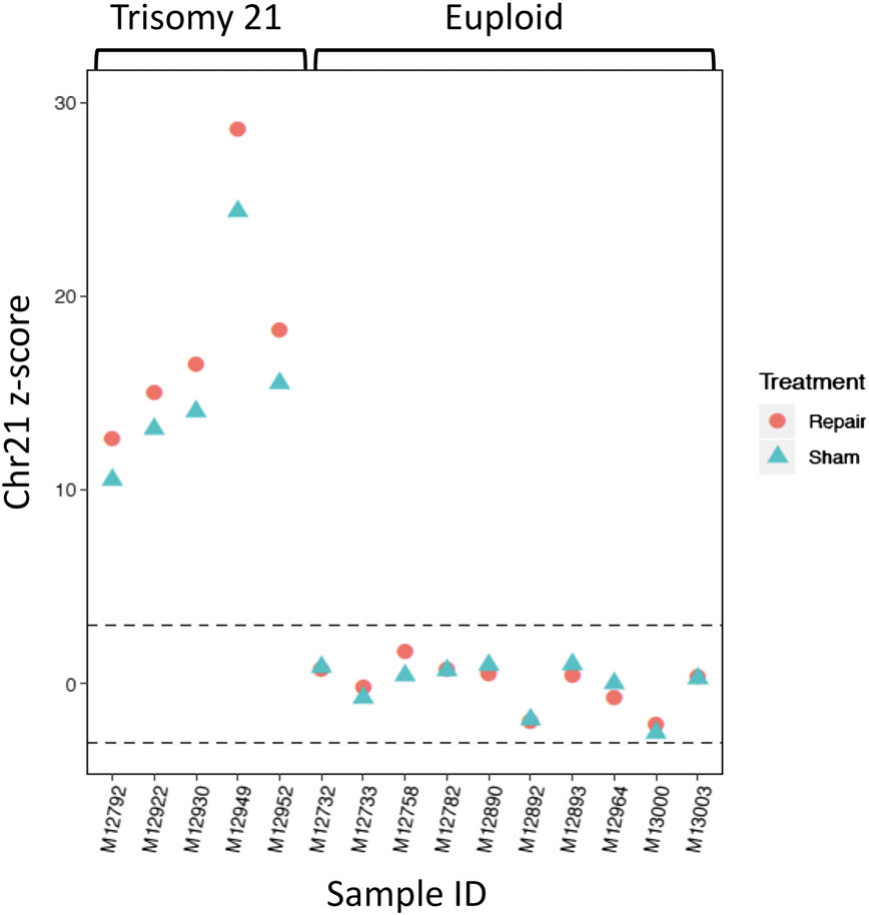
Improved noninvasive prenatal testing (NIPT) performance of trisomy 21 after PreCR repair treatment. Chromosome 21 *z* scores of five trisomy 21 (left) and 10 euploid (right) samples were compared. Repaired trisomy 21 samples showed increased *z* scores and better separation from the euploid samples [Colour figure can be viewed at wileyonlinelibrary.com]

As the repaired cfDNA sample has more intact long cfDNA molecules that are analyzable from the fetus, we aimed to demonstrate its potential application for prenatal diagnosis. We recruited five pregnancies with trisomy 21 fetuses and compared the chromosomal representation of Chr21 with the 10 first trimester euploid cases, which would be the most clinically relevant samples for NIPT. We observed that after PreCR repair treatment, Chr21 *z* scores for the trisomy 21 cases showed an average increase in 17.3% (ranging from 14.4% to 19.8%) and exhibited better separation from the *z* scores of the euploid samples (Figure 6). These results suggested that PreCR repair treatment may improve NIPT performance of trisomy 21 pregnancies.

## 4 DISCUSSION

Our findings demonstrated that cfDNA repair using the PreCR repair mix could recover a subset of long cfDNA molecules in maternal plasma. Relative enrichment of long cfDNA molecules after repair over short ones suggests that long cfDNA molecules contain more reparable DNA damages than their short counterparts. Given that long cfDNA molecules (eg, greater than 250 bp) existed in the maternal circulation in trace amounts, any enrichment by repair might result a significant increment in relative terms. Indeed, our data from the Illumina and Pacific Biosciences SMRT sequencing platforms illustrated enrichments of 68.8% and 31.3%, respectively, of cfDNA molecules longer than 250 bp. The enrichment became even more notable when one focused on even longer cfDNA molecules. For example, for cfDNA molecules between 450 and 600 bp in size, the enrichment amounts were, respectively, 109.4% and 44.3%, as measured using the Illumina and the Pacific Biosciences SMRT platforms. The variations of enrichment amounts by the two sequencing platforms were due to the fundamental differences between their sequencing methodologies and data output. Illumina and Pacific Biosciences SMRT platforms are based on different principles resulting in different functional specifications. Illumina sequencing is a form of second‐generation sequencing and performs DNA sequencing on an amplified cluster of DNA molecules. Pacific Biosciences sequencing is a form of third‐generation sequencing and performs sequencing on a single DNA molecule in a well. The Illumina platform only analyzes short DNA library molecules (usually up to 600 bp) and has higher sequencing output. In contrast, SMRT platform can sequence a DNA fragment up to 20 to 30 kb in length, but it analyzes far fewer DNA molecules per run. As the SMRT experiments had been performed on pooled samples, actual sequencing output obtained for each sample was low, but it allows the assessment of PreCR repair treatment on long cfDNA molecules. For cfDNA molecules longer than 600 bp as measured by the Pacific Biosciences SMRT platform, the enrichment following DNA repair was 50.4%. In short, PreCR repair treatment could successfully recover long cfDNA molecules from maternal plasma. It appeared that the longer the cfDNA molecule was, the higher was the enrichment.

The PreCR repair mix we used in this study has been shown to repair a variety of DNA damages, including DNA nicks, gaps, modifications of DNA molecules such as oxidation, and deamination, loss of DNA bases, and pyrimidine‐dimer formation.^26^, ^27^ In forensics, it was reported that the PreCR repair mix could restore short tandem repeat profiles from UV‐damaged DNA samples^26^ and artificially degraded DNA, mimicking the exposure of native DNA to oxidizing agents, hydrolytic conditions, and ionizing radiation.^27^ In archeology, it was used to recover heavily damaged ancient DNA samples.^28^ A similar repair procedure was applied in molecular diagnostics using formalin‐fixed paraffin‐embedded samples.^29^ However, this repair mix could not repair damages such as dsDNA fragmentation and DNA‐protein cross‐linking.

It is possible that different mechanisms might be at play for the generation of short and long cfDNA molecules in plasma. For example, cfDNA molecules shorter than 200 bp would likely be produced by enzymatic digestion during apoptosis. On the other hand, long (greater than 250 bp) cfDNA molecules would likely be generated by other cell death mechanisms, such as necrosis.^21^, ^37^, ^38^, ^39^, ^40^ As discussed earlier, the enrichment of cfDNA molecules by PreCR repair was more prominent between 250 and 600 bp. The latter lengths are reminiscent of di‐ and tri‐nucleosomal patterns (approximately 330 and 500 bp, respectively). From our data, the enrichment of long but not short cfDNA molecules after repair suggested that most of the reparable DNA damages might originate from cell death mechanisms other than apoptosis (eg, necrosis). In contrast, an absence of short cfDNA molecule enrichment after PreCR repair treatment suggested that such short cfDNA molecules might not contain reparable DNA damages.

Breaking down the total cfDNA into fetal and maternal fractions, our data suggested that DNA damages are not randomly distributed throughout cfDNA molecules of different origins. Although our data revealed that long cfDNA enrichments were occurring in both fetal and maternal cfDNA molecules, the magnitude of enrichment of long fetal cfDNA molecules (82.3%) was higher than that for cfDNA molecules of maternal origin (66.7%) (Figure 2). In line with this, the small but consistent overall fetal fraction increment after repair suggested that fetal cfDNA molecules might bear a higher percentage of reparable DNA damages (Figure 3). Possible reasons for this observation could be due to higher accessibility of fetal cfDNA molecules by DNA‐cutting enzymes.^41^ The relatively lower enrichment (14.7%) of long fetal cfDNA molecules following DNA repair in target captured libraries comparing with noncaptured enrichment (89.0%) could be due to competition effect between DNA templates with different lengths during target probes hybridization. For example, one particular case (M12732) demonstrated long cfDNA fragments enrichment (43.9%, from 2.5% to 3.6%) after PreCR repair treatment (Figure 1A,B). However, this enrichment became marginal (2.5%, from 6.0% to 6.2%) in the target capture setting (Figure 4B). We believe that this was due to capture efficiency differences between different samples.

The ability of the PreCR repair mix to restore damaged DNA opens up many potential applications. In prenatal diagnosis, our data demonstrated that PreCR repair treatment may contribute to improved NIPT performance for trisomy 21 screening (Figure 6). *z* scores of repaired trisomy 21 samples were better separated from those of the euploid samples (Figure 6). This is especially important for a case where the Chr21 *z* score is borderline and close to the euploid‐aneuploid cutoff without repair treatment. Future investigation is needed to investigate the performance of this approach in larger scale studies and if such improvement can be extended to the detection of other chromosomal aneuploidies. Beyond enhancing the statistical confidence of discrimination of euploidy and aneuploidy, repair of cfDNA may improve the call rate of NIPT. In particular, scenarios with nonreportable NIPT results caused by insufficient fetal DNA fraction^42^, ^43^, ^44^, ^45^, ^46^ or poor cfDNA quality might be improved by DNA repair. Examples of such scenarios might include NIPT in very early pregnancies and samples obtained from pregnant women with high body mass indices.^12^, ^47^, ^48^, ^49^ Furthermore, the ability of cfDNA repair to reveal more long analyzable cfDNA molecules in plasma might open up the possibility of using NIPT for long genomic targets, eg, sequences involved in triplet repeat disorders such as the fragile X syndrome (FXS). In FXS, the length of the CGG tandem repeats in patients with fully mutated *FMR1* alleles^50^, ^51^ is longer than 600 bp.

In conclusion, this study has revealed a preferential recovery of long cfDNA molecules in maternal plasma after PreCR repair treatment. Small but consistent increment of overall fetal DNA fraction is contributed by higher fetal‐derived cfDNA molecules enrichment. Practically, this PreCR repair treatment is a convenient single‐step procedure with low unit cost (~$10 per reaction). We hope that the data presented here might catalyze further research to translate these observations into clinical enhancements in NIPT.

## CONFLICTS OF INTEREST

K.C.A.C., R.W.K.C., and Y.M.D.L. are cofounders of the DRA Company Limited and have licensing arrangements with Illumina, Sequenom, Xcelom, and DRA. J.S.L.V., P.J., J.C.H.T., R.W.K.C., and Y.M.D.L. have filed a patent application on aspects of this work.

## FUNDING INFORMATION

This work was supported by the Hong Kong Research Grants Council Theme‐Based Research Scheme (T12‐403/15‐N). Y.M.D.L. is supported by an endowed chair from the Li Ka Shing Foundation.

## Supporting information

Table S1. Subjects information and sequencing data summary.

Figure S1. A. Size profile comparison of sham‐ and PreCR‐repaired cfDNA in log scale representation from a representative third trimester maternal plasma (M12778). B‐D. Averaged size profile comparison of sham‐ and PreCR‐repaired cfDNA of all 20 samples (B), ten first trimester samples only (C) and ten third trimester samples only (D) maternal pregnancies. Enrichments in long (>250 bp) cfDNA molecules in repaired sequencing library is prominent.
Figure S2. A‐B. Size profile comparison of sham‐ and PreCR‐repaired cfDNA in log (A) and linear (B) scale representation. For A, averaged size profiles of fetal (upper panel) and maternal (lower panel) cfDNA molecules, from ten first trimester samples only, ten third trimester samples only and all 20 samples, are represented. For B, lower panels are the magnified size profiles of long cfDNA molecules (black boxes).
Figure S3. A‐B. Size fractionated fetal DNA fractions of first (A) and third (B) trimester maternal plasma cfDNA (10 samples each) after sham or PreCR repair treatment. Notice the mild increase of fetal DNA fractions in long (approx. 300 bp) cfDNA molecules in repaired groups.
Figure S4. Enrichment of long cfDNA molecules after PreCR repair treatment in target captured libraries. Targeted capture probes were hybridizing on sequences of Chr6 (both mother and fetus) and ChrY (fetus only). Averaged size profile comparison of sham‐ and PreCR‐repaired cfDNA from pooled first and third trimester maternal plasma (4 samples each). Lower panels are the magnified size profiles of long cfDNA molecules (a: 250–450 bp; b: 400–600 bp).
