# Supplementary material for: Enrichment of fetal and maternal long cell‐free DNA fragments from maternal plasma following DNA repair
Source: Prenat Diagn. 2019 Jan 10;39(2):88–99. doi: 10.1002/pd.5406 (PMC6619283; doi:10.1002/pd.5406)
Supplement: Supplementary file 1 — Table S1. Subjects information and sequencing data summary. [file PD-39-88-s001.docx]

**Supplemental information**

Figure S1. A. Size profile comparison of sham- and PreCR-repaired cfDNA in log scale representation from a representative third trimester maternal plasma (M12778). B-D. Averaged size profile comparison of sham- and PreCR-repaired cfDNA of all 20 samples (B), ten first trimester samples only (C) and ten third trimester samples only (D) maternal pregnancies. Enrichments in long (>250 bp) cfDNA molecules in repaired sequencing library is prominent.

Figure S2. A-B. Size profile comparison of sham- and PreCR-repaired cfDNA in log (A) and linear (B) scale representation. For A, averaged size profiles of fetal (upper panel) and maternal (lower panel) cfDNA molecules, from ten first trimester samples only, ten third trimester samples only and all 20 samples, are represented. For B, lower panels are the magnified size profiles of long cfDNA molecules (black boxes).

Figure S3. A-B. Size fractionated fetal DNA fractions of first (A) and third (B) trimester maternal plasma cfDNA (10 samples each) after sham or PreCR repair treatment. Notice the mild increase of fetal DNA fractions in long (approx. 300 bp) cfDNA molecules in repaired groups.

Figure S4. Enrichment of long cfDNA molecules after PreCR repair treatment in target captured libraries. Targeted capture probes were hybridizing on sequences of Chr6 (both mother and fetus) and ChrY (fetus only). Averaged size profile comparison of sham- and PreCR-repaired cfDNA from pooled first and third trimester maternal plasma (4 samples each). Lower panels are the magnified size profiles of long cfDNA molecules (a: 250-450 bp; b: 400-600 bp).

Table S1. Subjects information and sequencing data summary.
